# Clinical outcomes following switching antipsychotic treatment due to market withdrawal: a retrospective naturalistic cohort study of pipotiazine palmitate injection (Piportil Depot) discontinuation, subsequent acute care use and effectiveness of medication to which patients switched

**DOI:** 10.1177/20451253211067042

**Published:** 2022-01-30

**Authors:** Rollo J.G. Sheldon, Marco Pereira, George Aldersley, Tim Sales, Jed Hewitt, Ray Lyon, Richard Whale

**Affiliations:** Sussex Partnership NHS Foundation Trust, Brighton, UK; Brighton and Sussex Medical School, Brighton, UK; Faculty of Psychology and Educational Sciences, University of Coimbra, Coimbra, Portugal; Brighton and Sussex Medical School, Brighton, UK; Sussex Partnership NHS Foundation Trust, Brighton, UK; Sussex Partnership NHS Foundation Trust, Brighton, UK; Sussex Partnership NHS Foundation Trust, Brighton, UK; Sussex Partnership NHS Foundation Trust, The Aldrington Centre, 35 New Church Road, Brighton BN3 4AG, UK; Sussex Partnership NHS Foundation Trust, Brighton, UK; Brighton and Sussex Medical School, Brighton, UK

**Keywords:** antipsychotic, atypical, depot, discontinuation, dopamine antagonist, long acting injection, market withdrawal, piportil, pipothiazine, pipotiazine, switch, typical

## Abstract

**Introduction:**

Pipotiazine palmitate depot injection (Piportil) was withdrawn from the UK marketplace in 2015. Few studies exist on the clinical impact of such market withdrawal. **Purpose:** We aimed to identify a cohort of patients switching from pipotiazine following this withdrawal and explore factors associated with effectiveness of the medication switched to and subsequent acute service use.

**Methods:**

A naturalistic retrospective cohort study was conducted in Sussex, United Kingdom. Those discontinuing pipotiazine solely due to market withdrawal were identified from electronic patient database and manual searching. Multivariate logistic regression analyses and survival analyses were performed to explore associations between available baseline variables and dichotomous all-cause discontinuation of the next prescribed medication and admission to acute mental health services over the subsequent year.

**Results:**

Of 205 patients identified as receiving pipotiazine in October 2014, 137 switched from this due to market withdrawal. Over the subsequent year, 31.5% discontinued the medication to which they were switched and 19% required acute care. Drug class switched to (typical depot vs atypical long acting injection (LAI) vs atypical oral) had no significant association with discontinuation. Switch to atypical LAI was significantly associated with acute care in comparison to typical depot. Those with a schizophrenia diagnosis were significantly less likely to discontinue switched medication or to receive acute care in comparison to those with schizoaffective disorder. Women were significantly more likely to discontinue switched medication than men. Of those requiring acute care, only 38% had required this in the previous 2 years.

**Conclusions:**

Antipsychotic market withdrawal has demonstrable negative clinical implications and requires careful clinical management. Increased acute care rates in those receiving an atypical LAI versus a typical depot following pipotiazine suggests lower effectiveness or possible withdrawal effects. No significant difference between depots, LAIs and oral medications on discontinuation supports the importance of a collaborative, fully informed approach when deciding next treatment options.

## Introduction

Pipotiazine is a first-generation phenothiazine antipsychotic with primary affinity for the dopamine D2 receptor and was granted a UK licence for the treatment of schizophrenia in 1980. In March 2014, the French Health Authorities (L’Agence nationale de sécurité du médicament et des produits de santé) suspended the Good Manufacturing Certificate for the supplier (La Somet) of the active ingredient for pipotiazine palmitate depot injection (Piportil) due to ‘potential contamination issues’ (personal correspondence from Sanofi, the manufacturer of pipotiazine depot, 5/2/18). In October 2014 Sanofi, advised UK clinicians to switch patients to alternative treatments and the product was formally withdrawn from the marketplace in March 2015. Psychotropic medications have most frequently been withdrawn from the UK marketplace in recent years due to a late identified poor risk/benefit profile, for example, droperidol and thioridazine.^1^ Little evidence of clinical outcomes of antipsychotic marketplace withdrawal is available in the published literature. Only one previous naturalistic report of switching from pipotiazine depot was identified, albeit with a small sample size: four of a total of 17 patients were admitted to hospital in the 12 months after switching, including the only three who had chosen to switch to an oral medication.^2^ Purhonen *et al.*^3^ used the Finnish national patient registry to evaluate schizophrenia patient outcomes following the earlier withdrawal of the oral dopamine D2 antagonist thioridazine, reporting that the rate of hospital admission and hospitalisation days in discontinuers markedly increased and these patients were exposed to an unacceptable greater risk of relapse. Demers *et al.*^4^ reported a chart review following a lack of availability of haloperidol decanoate in Canada and also identified a subsequent clear increase in hospitalisation rate. Zanker and Ferraro^5^ reported a series of 9 admissions to hospital following market withdrawal of trifluoperazine and fluphenazine in Australia.

Aside from studies of full medication discontinuation, other antipsychotic switching studies are primarily concerned with outcomes of switching *to* a specific medication, rather than *from* it (such as the majority of new antipsychotic licencing studies) and require an adequate clinical reason for switching. In the naturalistic CUtLASS trials, for example, patients who experienced *inadequate efficacy* or *adverse effects* on an existing antipsychotic were randomly switched to a typical or atypical antipsychotic of the clinician’s choice; those switching from an antipsychotic depot preparation had a poorer outcome when they were switched to an oral antipsychotic other than clozapine, which appeared partly explained by medication adherence.^6^ When considering discontinuation of antipsychotics that have been switched to, randomised studies report consistently lower rates than purely naturalistic studies, so the latter importantly contribute to the wider literature to better reflect real life clinical outcomes.^7^

Recommendations for antipsychotic switching are available and more recently have primary focus on collaborative decision making with the recipient around tolerability and efficacy information of different preparations.^8,9^ No national guidance on optimal strategy following pipotiazine depot withdrawal was offered but informed recommendations were published by Haddad *et al.*^10^ Medication shortages in psychiatry and a lack of clear alternatives of similar pharmacology have been increasingly reported internationally and market withdrawal of other currently used long acting specific D2 antagonist injections may become a reality.^4^

The aim of this study was to naturalistically explore optimal switching strategies from pipotiazine, at the time of its market withdrawal. The primary outcome was the impact of antipsychotic group (typical vs atypical and oral vs long acting injection) on whether the drug switched to was discontinued and the secondary outcome was whether acute psychiatric care was utilised, both outcomes within a year from switch date. Time to naturalistic all-cause discontinuation of an antipsychotic medication appears to be a reasonable marker of its effectiveness, incorporating patient and clinician aspects, acceptability and efficacy, and used extensively as an outcome in previous studies.^11^ A medication that is more acceptable to take, with fewer adverse effects and perceived beneficial effects by the recipient as well as the prescriber is likely to be taken for a longer period of time and on balance equate to greater effectiveness. In comparison with the majority of previous switching studies, this study importantly differed as switch did not occur because of adverse effects, ineffectiveness or doctor or patient choice.

## Methods

A naturalistic retrospective cohort study design was adopted to examine clinical outcome of pipotiazine market withdrawal and to explore which medication group may offer optimal effectiveness after switching. All patients identified as receiving pipotiazine depot injection in October 2014 under the care of Sussex Partnership NHS Foundation Trust mental health services, covering Sussex, United Kingdom, were eligible for this study. Sussex has a population of approximately 1.4 million, including areas with both low and high social deprivation indices. Estimates of numbers of people receiving depot or long acting antipsychotic medication are not available due to the wide diversity of prescribers in both primary and secondary care.

Patients receiving pipotiazine were identified from depot clinic lists, email requests to clinicians and electronic search of all clinical records held at the Trust (search item ‘pipo*’ identified in electronic records from October 2014). Those who were receiving pipotiazine in October 2014 and were switched from this within the following 12 months solely due to market withdrawal were the study sample. Patients were excluded if they discontinued pipotiazine for any other reason. The small group of patients who had been identified by clinicians to be prioritised to continue receiving stockpiled pipotiazine beyond October 2015, for whatever clinical reason, were also excluded. If larger and not clinically different, this group may have provided a useful control in this study. The study team had no influence on which preparation was switched to and this decision was solely between the recipient and their clinician. Baseline data recorded at the time of switching from pipotiazine were gender, age at switch, primary psychiatric diagnosis, duration of illness before switch, duration of pipotiazine depot treatment, 4 weekly pipotiazine dose, switch setting (inpatient or outpatient) and the antipsychotic the patient was switched to, if any. If time durations were of many years and case records not fully clear, the most conservative clear evidence available was used and agreed between two authors. Naturalistic clinical outcomes within 1 year of switching with reliable recording in clinical records were chosen. The primary dichotomous outcome was whether or not patients had remained on the new medication they were switched to at 12 months following switch. The secondary dichotomous outcome was whether patients had required acute psychiatric services, as defined by either admission to hospital or being under the care of the Crisis Response and Home Treatment Team within 12 months from switch. Time to both of these outcomes from the switch date was also identified. In the group receiving acute care following pipotiazine discontinuation, episodes of acute care in the 2 years prior to discontinuation were also counted, for rough comparison.

Data were analysed using SPSS version 25. Logistic regression analysis was adopted to identify influence of baseline variables on the primary and secondary outcomes. Kaplan-Meier survival analysis was performed to explore the times to discontinuation of medication group switched to. Medication groups were predefined as ‘typical depot’, ‘atypical long-acting injection’ (LAI), ‘typical oral’, ‘atypical oral’ or clozapine. The terms ‘typical’ and ‘atypical’ were adopted for simplicity in this study but importantly reflect older medications with primary dopamine D2 antagonist activity and newer medications with more complex actions including serotonin antagonism, respectively. Ethical conduct of the study was approved by the Sussex Partnership NHS Foundation Trust audit governance committee on 14th November 2016 as not requiring individual patient consent. Data was collected prior to April 2018. STROBE reporting guidelines were adopted for this manuscript (https://www.strobe-statement.org/).

## Results

**Figure 1.**
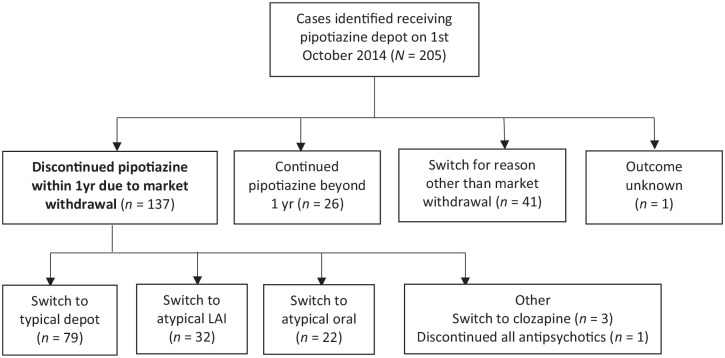
Cohort recruitment flow chart.

Following electronic case record search and scrutiny of individual records, 205 cases were identified as receiving pipotiazine depot at the start of October 2014 in Sussex (see Figure 1). As the primary study group, 137 were clearly switched from this due to market withdrawal within the following year. One patient rapidly discontinued all medication and is not described further for information governance reasons. Twenty-six patients continued beyond one year on stockpiled medication. Reasons for stopping pipotiazine other than market withdrawal were patient refusal (including non-adherence), adverse effects and lack of an adequate treatment response. Three patients died during the study period for reasons which appeared independent of switching from pipotiazine, on consensus review by two authors. None of these were experiencing an apparent relapse of psychosis. However, long term adverse physical consequences of antipsychotic use may be contributing factors in these cases.

Demographic features and recorded variables of the primary study group are shown in Table 1 and sub-divided by switch medication group. The greatest proportion of this group were male (65%), over 40 years old (76%), had a schizophrenia spectrum diagnosis within ICD10 F20-29 (82%), had a duration of illness over 10 years (64%), had been receiving pipotiazine for over 5 years (52%) and were switched from pipotiazine in the community (as opposed to acute care) (88%). Only one patient had a duration of illness of less than one year and only two had been receiving pipotiazine for less than one year. The majority of patients were switched to a typical depot injection (flupenthixol 59, fluphenazine 7, haloperidol 7 or zuclopenthixol 6), followed by an oral antipsychotic (amisulpride 2, aripiprazole 18, olanzapine 5, quetiapine 4, risperidone 3, sulpride 1 and clozapine 3) and then an atypical LAI (aripiprazole 7, paliperidone 13, risperidone 1). For the purposes of analysis in this study, sulpride was considered an atypical oral antipsychotic and included in that group. No typical oral antipsychotics were therefore used. Clozapine was retained as a separate group. Comparing baseline characteristics between drug groups, only the clozapine group significantly differed in having a lower mean age and a greater proportion of inpatient initiations. The rates of those discontinuing switch medication and receiving acute care within the following year are also detailed in Table 1.

**Table 1.** Baseline characteristics and outcomes of cohort discontinuing pipotiazine palmitate. Total sample and division by switch drug group.

	Typical depot	Atypical LAI	Oral antipsychotic	Clozapine	Total (*n* = 137)^a^
Age at switch, mean (SD), years**	49.33 (13.20)	49.88 (12.82)	57.36 (14.94)	28.67 (1.53)	50.38 (13.85)
Gender (female/male)	23/56	15/17	8/14	1/2	47/89
Duration of illness > 10 years (yes)	47 (59.5%)	23 (71.9%)	14 (63.6%)	2 (66.7%)	87 (63.5%)
Duration of illness > 5 years (yes)	71 (89.9%)	28 (87.5%)	21 (95.5%)	2 (66.7%)	123 (89.8%)
Pipotiazine duration, ^b^ mean (SD), years	5.37 (3.35)	4.95 (5.96)	6.62 (2.87)	5.00 (1.73)	5.49 (4.03)
4 weekly dose, mean (SD), mg	86.89 (44.06)	82.03 (33.74)	86.82 (54.78)	150.00 (86.60)	87.46 (45.34)
Diagnosis					
Bipolar disorder	4 (5.1%)	1 (3.1%)	3 (13.6%)	—	8 (5.8%)
Depression disorder	3 (3.8%)	—	—	—	3 (2.2%)
Organic psychosis	1 (1.3%)	—	1 (4.5%)	—	2 (1.5%)
Personality Disorder	6 (7.6%)	2 (6.3%)	3 (13.6%)	1 (33.3%)	12 (8.8%)
Schizophrenia	51 (64.6%)	22 (68.8%)	11 (50.0%)	2 (66.7%)	87 (63.5%)
Schizoaffective disorder	12 (15.2%)	7 (21.9%)	4 (18.2%)	—	23 (16.8%)
Other Schizophrenia spectrum disorder	2 (2.5%)	—	—	—	2 (1.5%)
Switch setting – Community (vs. Inpatient)*	72 (91.1%)	28 (87.5%)	18 (81.8%)	1 (33.3%)	120 (87.6%)
Discontinuation by 1 year (yes)^c^	19 (25.7%)	12 (38.7%)	8 (38.1%)	1 (33.3%)	41 (31.5%)
Acute service involvement within 1 year (yes)	11 (13.9%)	10 (31.3%)	4 (18.2%)	—	26 (19.0%)

LAI, long acting injection.

a Includes 1 case who discontinued all medication.

b Information unavailable in 1 case.

c information unavailable in 7 cases.

** *p* < .01; *p < .05.

### Discontinuation of switch medication

**Figure 2.**
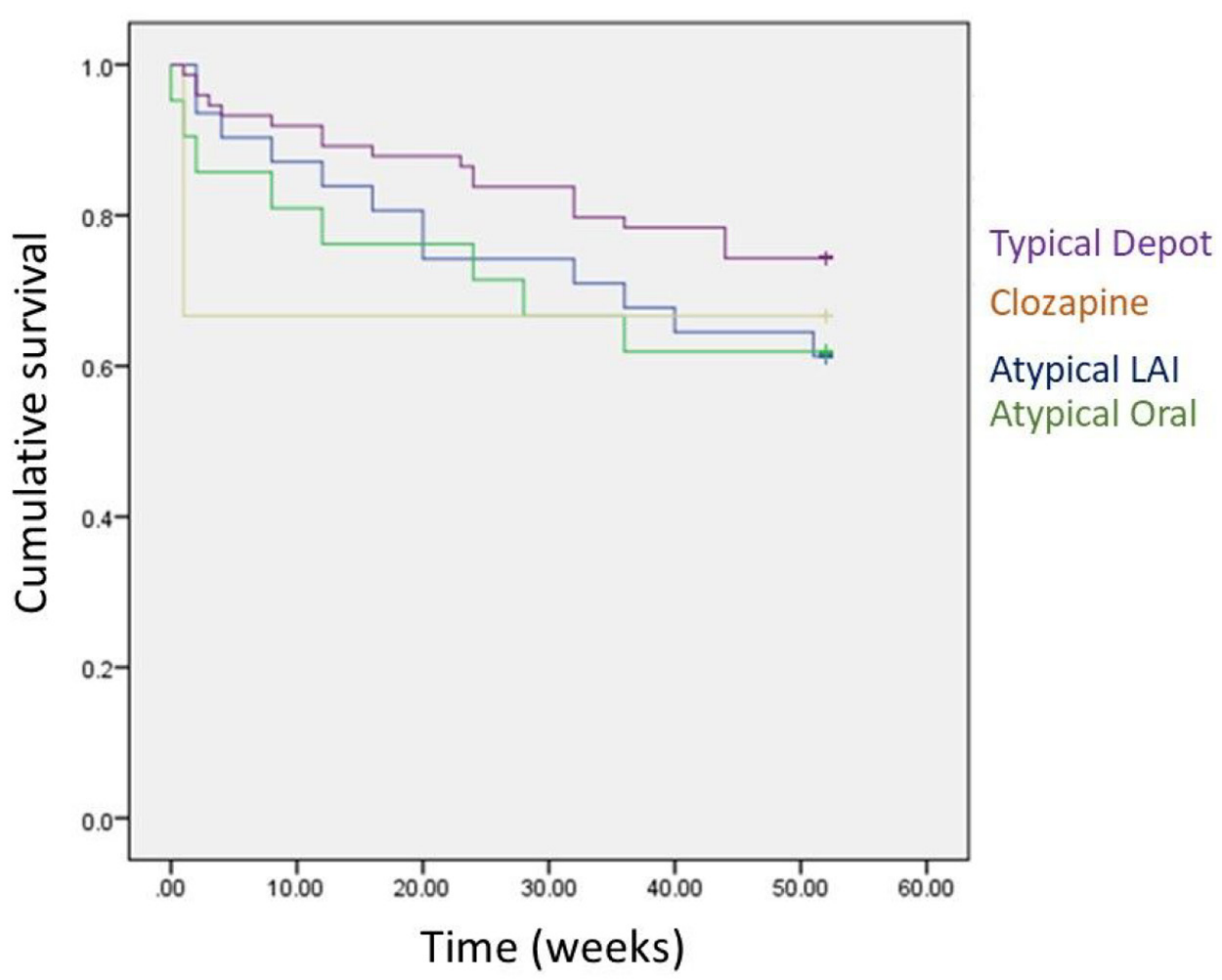
Kaplan-Meier survival plot of remaining on medication to which they were switched from pipotiazine palmitate.

By 12 months, 41 patients (32%; 7 missing outcome values) had discontinued switch medication. Mean (*SD*) time to discontinuation in this group was 18.6 (15.1) weeks. The group switched to a typical depot had the lowest discontinuation rate (Table 1). Mean (*SD*) time to discontinuation by drug group was typical depot 20.8 (15.4) weeks, atypical LAI 20.25 (16.20), atypical oral 13.9 (13.8) and only one person discontinued clozapine, shortly after initiation. Logistic regression analysis of influence of baseline variables on switch medication discontinuation by 1 year revealed no significant impact of the medication switch group but a significant impact of gender (women were more likely to discontinue than men, *p* = 0.016). While diagnosis had no significant effect overall (*p* = 0.070), those with a schizoaffective disorder diagnosis were more likely to discontinue when compared to those with schizophrenia (*p* = 0.038), as shown in Table 2. No significant gender differences by diagnosis were observed. Kaplan-Meier survival analysis of switch medication discontinuation also revealed no overall significant influence of medication group on discontinuation (for Mantel-Cox Log Rank test, Chi-square = 2.65, *df* = 3, *p* = 0.449; Figure 2) or on pairwise comparisons between any switch groups (all *p* > 0.100).

**Table 2.** Logistic regression analysis of influence of baseline factors on discontinuation of antipsychotic medication to which they were switched, by one year.

	Odds ratio [95% CI]	*p*
Age at switch (years)	1.00 [0.98-1.03]	.759
Gender (male vs. female)	2.58 [1.19-5.58]	.016
Duration of illness > 10 years (no vs. yes)	1.40 [0.64-3.06]	.404
Duration of illness > 5 years (no vs. yes)	0.71 [0.22-2.32]	.573
Pipotiazine duration^a^ (years)	0.95 [0.86-10.6]	.358
4 weekly dose (mg)	1.01 [1.00-1.01]	.256
Diagnosis		.070
Schizophrenia vs. Bipolar	2.25 [0.47-10.88]	.313
Schizophrenia vs. Personality Disorder	3.60 [1.00-13.02]	.051
Schizophrenia vs. Schizoaffective disorder	2.75 [10.6-7.15]	.038
Antipsychotic class switched to		.314
Typical depot vs. Atypical LAI	1.83 [0.75-4.56]	.185
Typical depot vs. Atypical oral	1.78 [0.64-4.96]	.269
Switch setting (Community vs. Inpatient)	0.89 [0.29-2.72]	.840

CI, confidence interval; LAI, long acting injection.

a Information unavailable in 1 case.

### Acute care following switch

**Figure 3.**
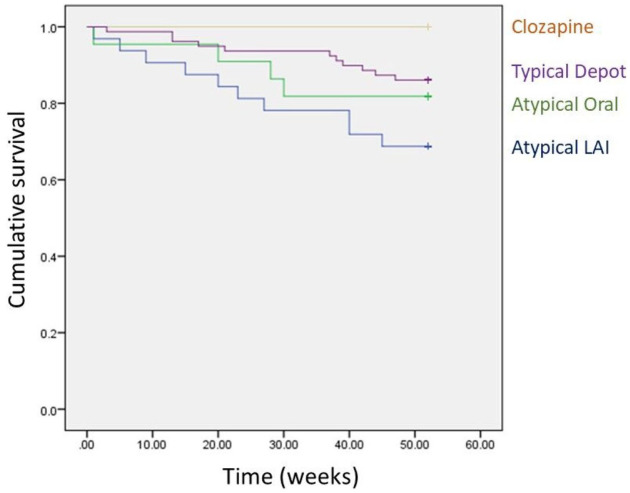
Kaplan-Meier survival plot of remaining without acute care following switch from pipotiazine palmitate.

Twenty-six patients (19% of the cohort) required acute care in the year following switch from pipotiazine. Those remaining in the same rehabilitation hostel, nursing home or forensic unit over the year (*n* = 16) were deemed as not having a new acute care period. Mean (*SD*) time to acute care (in those requiring it) was 24.3 (14.8) weeks. Mean (*SD*) time to acute care by drug group was: typical depot 28.6 (15.4) weeks, atypical LAI 22.5 (15.5), atypical oral 19.8 (13.2). No one in the small clozapine group required acute care. Those who switched to typical depot had the next lowest proportion of acute care episodes (Table 1). Logistic regression analysis of influence of baseline variables on acute care requirement is shown in Table 3. A significant difference was observed between those switched to typical depot or atypical LAI, with the former being significantly less likely to require acute care over the year (11 out of 68 (16%) vs 10 out of 22 (45%) respectively; *p* = 0.039). Again, while no overall effect of diagnosis is observed, a significant difference between schizophrenia and schizoaffective disorder was observed, with those having schizophrenia being less likely to receive acute care (*p* = 0.025). Kaplan-Meier survival analysis of acute care requirement also revealed no significant influence of overall medication group (for Mantel-Cox Log Rank test, Chi-square = 5.60, *df* = 3, *p* = 0.133; Figure 3). For pairwise comparisons between switch groups however, a significant difference was corroborated between typical depots and atypical LAIs (for Mantel-Cox Log Rank test, Chi-square = 4.85, *p* = 0.028). Consistent with the logistic regression analysis, the only significant pairwise difference for diagnosis on survival was for schizophrenia vs schizoaffective diagnosis (Mantel-Cox Log Rank test, Chi-square = 5.86, *p* = 0.015). A highly significant association was observed between discontinuing switch medication and requiring acute care (Pearson chi square = 36.5, *p* < 0.001).

**Table 3.** Logistic regression of influence of baseline factors on acute service involvement within one year of switch from pipotiazine palmitate.

	Odds ratio [95% CI]	*p*
Age at switch (years)	0.99 [0.96-1.02]	.489
Gender (female vs. male)	1.79 [0.75-4.25]	.190
Duration of illness > 10 years (no vs. yes)	1.37 [0.55-3.43]	.501
Duration of illness > 5 years (no vs. yes)	1.46 [0.31-6.94]	.638
Pipotiazine duration^a^ (years)	1.04 [0.94-1.15]	.471
4 weekly dose (mg)	1.01 [1.00-1.02]	.175
Diagnosis		.097
Schizophrenia vs. Bipolar	2.08 [0.38-11.55]	.401
Schizophrenia vs. Personality Disorder	3.13 [0.81-12.01]	.097
Schizophrenia vs. Schizoaffective disorder	3.33 [1.16-9.55]	.025
Antipsychotic class switched to		.117
Typical depot vs. Atypical LAI	2.81 [1.05-7.50]	.039
Typical depot vs. Atypical oral	1.37 [0.39-4.83]	.620
Switch setting (Community vs. Inpatient)	0.53 [0.11-2.49]	.424

CI, confidence interval; LAI, long acting injection.

a Information unavailable in 1 case.

Of the 26 patients requiring acute care, 6 of this group had required acute care in the year prior to pipotiazine discontinuation and 10 over the prior 2 years.

## Discussion

Few studies have explored the clinical outcomes of market withdrawal of antipsychotic medication. This is the first naturalistic switching study of relatively large sample size that we are aware of, to enable comparison of switch medication groups following market withdrawal of an antipsychotic depot injection. We attempted to identify all patients in secondary care receiving pipotiazine in our geographical area. We found a pipotiazine prescribing rate of approximately 146 per million population in Sussex, which is nearly twice the rate identified in Scotland by Haddad *et al.*^10^ The sub group of those receiving pipotiazine who were clinically stable, and would have remained on it if it had not been withdrawn, had notable chronicity of illness of various diagnostic groups (including those not falling within the pipotiazine treatment licence) and had largely been receiving this preparation for several years. Of this study sample (*n* = 137), 31.5% discontinued the medication switched to from pipotiazine over first year and 19% required acute care. Considering the primary aims of this study, drug class switched to had no significant association with discontinuation by 12 months. A significant association of switch to atypical LAI with requirement for acute care in the following year, in comparison to typical depot switch, was identified. A consistent significant influence on both primary outcomes was observed between schizophrenia and schizoaffective disorder diagnostic groups: those with schizophrenia were less likely to discontinue switched medication or to receive acute care. Women were significantly more likely to discontinue switched medication than men.

### Medication groups

The largest proportion of patients were switched from pipotiazine to a typical antipsychotic depot medication, most commonly flupentixol. The clinical rationale for this may have included similarity of mode of action (primary D2 receptor antagonism) and lower cost than newer alternatives. No one was switched to an oral typical antipsychotic but a notable proportion switched to an oral atypical (more than an atypical LAI), predominantly oral aripiprazole.

The process of choice of switch medication was not possible to explore using the methodology adopted but being given all switch options in a collaborative treatment manner may aid taking individual responsibility for preventing relapse and adherence to treatment. It is possible that those switching to oral medication were more likely to have had such a collaborative treatment discussion, which may have enabled a lower likelihood of relapse. The equivalence of outcome of switch to oral or depot/LAI preparation is important and inconsistent with previous findings.^2,6^ Differences in baseline variables between switch groups (excluding clozapine) were not evident, including site of initiation (those initiated as an inpatient may be deemed more severely unwell).

Switch to clozapine in an apparently clinically stable group is possibly surprising but they were few overall. Acute care initiation of clozapine is common due to the physical monitoring required and it is encouraging that this group was significantly younger, highlighting an early clinical identification of treatment resistance. Further outcome comparisons with this group are not possible due to small sample size.

### Rate of discontinuation

The rate of all-cause discontinuation of medication switched to, at 32% of the cohort by 1 year, was similar to previous studies of 1 year naturalistic discontinuation rates of newly initiated LAIs in the same geographical area, despite reason for switch in these other studies being largely due to prior antipsychotic intolerance, inefficacy or poor adherence.^7,12^ This finding suggests that clinical stability at baseline does not influence rate of all-cause discontinuation of medication subsequently switched to. Discontinuation rates vary between wider study groups and are notably lower in randomised studies, supporting the importance of naturalistic studies with large treatment groups.^7^

A lack of overall impact of drug switch group on discontinuation rate may be interpreted as equal effectiveness of all antipsychotics in such a setting,^12^ particularly notable when comparing oral vs depot and LAI preparations.

A similar attrition rate over the year was observed for the three main medication groups although oral antipsychotics tended to be discontinued earlier. While oral antipsychotics are deemed to have poorer adherence than depots and LAIs, the overall recorded discontinuation rate over the year was, importantly, not significantly different between groups. Numerically, survival was greatest in the group switched to a typical depot. Why women discontinued switched medication significantly earlier than men is unclear and not mediated by other variables identified in this study. This may have consistency with women being less likely to be adherent to cardiovascular treatments.^13,14^ While not directly comparable, Covell *et al.*^15^ explored randomly switching to risperidone microsphere LAI or staying on typical depot (fluphenazine or haloperidol) and time to discontinuation over a year in a small group who had some inadequate response or adverse effects; those in the switch group were more likely to discontinue over this period. This may support patients staying on existing treatment if possible but firm conclusions on relative effectiveness are difficult to draw from this report.

No previous studies have identified a difference between schizophrenia and schizoaffective diagnoses on the outcomes explored in this study. Similarly, no studies of pipotiazine in schizoaffective disorder were identified. Consistency of this finding between outcomes in our study supports its validity and the importance of differentiating between these diagnostic groups. Pipotiazine was not reported to have any greater beneficial effects on depression scales in the two randomised studies that measured this, when compared with other depot antipsychotics.^16^ In a recent meta-analysis, the typical antipsychotics haloperidol and chlorpromazine had no effect on mood scales in those with schizophrenia, whereas atypical antipsychotics (except ziprasidone) had some overall effect.^17^ Flupenthixol has a UK licence for treatment of depressive illness however.^18^

The duration of use of pipotiazine and the duration of illness prior to switching had no bearing on either primary outcome, as consistent with previous studies.^19^

### Acute care

The few identified earlier studies suggest a clinically destabilising impact of antipsychotic market withdrawal with an increased subsequent hospitalisation rate.^2,3^ The rate of 19% of our cohort requiring acute care within a year appears high in an otherwise clinically stable group: only 6 (23%) of this acute care group had required such care in the previous year (38% in the previous 2 years). The identified significant difference between typical and atypical depot /LAI switch groups is also notable. A pharmacodynamic effect may explain this with lower D2 receptor potency of atypical antipsychotics potentially equating to reduced efficacy or a withdrawal or rebound effect.^20^ The mean time to acute care of 24 weeks is much later than expected to be clearly explained as a withdrawal effect however. The finding of no significant difference between oral atypical and typical depot however indicates a more complex explanation beyond pharmacodynamics, as discussed above. Those switched to an oral antipsychotic may have been deemed to be more clinically stable and less likely to need acute care, although site of initiation was not significantly associated with subsequent acute care (those initiated as inpatients being less clinically stable). The importance of an adequate sample size is highlighted when compared to the previous pipotiazine withdrawal study in which all three patients who switched to oral preparations were subsequently admitted to hospital.^2^ Stone *et al.*^21^ found an equivalent readmission and time to discontinuation rate in groups switched to typical or atypical depots/LAI within normal clinical practice so our finding may highlight a difference between clinically stable (our study) and groups switched due to inefficacy or intolerance. Acute care was earlier in the group receiving oral medication, likely reflecting the ease of discontinuing oral medication relative to injected preparations, but with no significant difference in overall rate vs the other groups. Potentially unidentified covert non-adherence with oral medications may be less important if acute care rates between oral and depot/LAI switch groups are not significantly different.

We were unable to accurately retrospectively explore the reasons for acute care referral in this group but relapse of illness with associated acute clinical risk is clearly most likely. Risk associated with mood disorder in schizoaffective disorder, such as suicidality,^22^ may explain the greater need for acute care in this group. As expected, discontinuation of switched medication and acute care requirement were significantly correlated and discontinuation time was on average earlier than acute care, suggesting the former may lead to the latter.

### Limitations

Naturalistic retrospective uncontrolled studies have innate bias but we aimed to identify real life outcomes in this study, using reliably recorded and accessible clinical information, which included a sample of adequate size to enable comparison of medication subgroup outcomes and baseline variables. While this is the largest cohort described of the outcome of market withdrawal of a depot antipsychotic medication, numbers in individual medication groups were low (aside from flupentixol), hence requiring categorisation into typical and atypical groups. While this does not address differing properties of medication within groups, a significant difference in acute care outcome was identified. A prospective randomised study would enable clearer efficacy comparisons of a smaller range of switch options but clinical outcomes would be biased more positively than in real world naturalistic studies.^7^

Arguably, the outcomes studied may have been independent of pipotiazine discontinuation, however we were able to identify the unexpected difference between medication group switched to and subsequent acute care rate. Within-study control groups of switching from other medications would be required to identify what may be more unique to switch from pipotiazine.

Those under more intensely supportive community services such as Assertive Outreach or Early Intervention, or inpatient residential care may need to meet a higher threshold of illness severity to receive acute care. Such services may identify severe clinical relapse earlier however and involve acute services more promptly if required. While acute service use is a reliable measure of severe illness relapse and increase of associated risks, regular symptom scales are required to more clearly define symptomatic relapse and potential treatment failure. We chose to include both inpatient admission and community crisis care together to lower the relapse threshold identification level and aim to identify more clinical relapses. Other factors may have been relevant to the outcomes measured including ethnicity, the role of psychosocial stress, adverse events, adverse effects of medications and substance abuse which were not reliably measured in this cohort. Prospective investigation would enable systematic recording of such mediating factors but sample size would be inherently reduced.

### Clinical implications

While the numbers of those discontinuing pipotiazine may now be low, due to some increased availability through parallel suppliers, this study has important wider implications for potential market withdrawal of other antipsychotic medications. Relapse within the following year appears greater than if staying on the same medication if clinically stable. Switching from a discontinued typical antipsychotic to another typical product appears the most favourable in terms of reducing acute service use and associated costs, although the lack of difference with oral medication requires further exploration.

With the absence of significant difference in discontinuation rate, collaborative treatment decision making is certainly supported and may have benefit on relapse likelihood. Careful weighing up of metabolic vs movement disorder adverse effects between atypical and typical preparations is clearly complex, but the latter when severe are arguably more socially disabling. Oral medications were at least of equal benefit on both outcomes measured so have an important place as switch options. Market withdrawal offers an important opportunity for treatment review which should also include open discussion of potential harms and benefits of ongoing medication use and the option of managed discontinuation of antipsychotic treatment, aligned to developing evidence, of which there were none in this study.

Further market withdrawals and resulting reduced availability of currently used typical antipsychotic preparations is likely; fluphenazine (Modecate) manufacture was discontinued in 2018 due to unpredictability of supply of the active ingredient from the global manufacturer. Our findings will aid development of recommendations for switching strategies in the future.
